# Increased Preoperative Plasma Level of Microbial 16S rDNA Translocation Is Associated With Relapse After Prostatectomy in Prostate Cancer Patients

**DOI:** 10.3389/fonc.2019.01532

**Published:** 2020-01-15

**Authors:** Tongwen Ou, Zejun Zhou, David P. Turner, Baoli Zhu, Michael Lilly, Wei Jiang

**Affiliations:** ^1^Department of Urology, Capital Medical University Affiliated XuanWu Hospital, Beijing, China; ^2^State Key Laboratory of Developmental Biology of Freshwater Fish, College of Life Sciences, Hunan Normal University, Changsha, China; ^3^Department of Microbiology and Immunology, Medical University of South Carolina, Charleston, SC, United States; ^4^Department of Pathology and Laboratory Medicine, Medical University of South Carolina, Charleston, SC, United States; ^5^CAS Key Laboratory of Pathogenic Microbiology and Immunology, Beijing Key Laboratory of Antimicrobial Resistance and Pathogen Genomics, Institute of Microbiology, Chinese Academy of Sciences, Beijing, China; ^6^Savaid Medical School, University of Chinese Academy of Sciences, Beijing, China; ^7^Department of Pathogenic Biology, School of Basic Medical Sciences, Southwest Medical University, Luzhou, China; ^8^Division of Hematology and Oncology, Department of Medicine, Medical University of South Carolina, Charleston, SC, United States; ^9^Division of Infectious Diseases, Department of Medicine, Medical University of South Carolina, Charleston, SC, United States

**Keywords:** prostate cancer, microbial translocation, prostatectomy, relapse, prostate-specific antigen

## Abstract

**Background:** The environmental factors for promoting prostate cancer (PCa) recurrence remain unknown.

**Methods:** A retrospective cross-sectional study was conducted in healthy men (*n* = 12) and PCa patients undergoing prostatectomy (*n* = 27). Plasma preoperative level of total cell-free bacterial 16S rDNA, a marker of microbial translocation, was evaluated by qPCR. Plasma levels of prostate-specific antigen (PSA) were evaluated by ELISA.

**Results:** Similar degrees of microbial translocation were found in healthy men and patients. However, the levels of microbial 16S rDNA were increased in patients with cancer relapse (*n* = 10) compared to patients without relapse (*n* = 17) after prostatectomy. Furthermore, the levels of microbial 16S rDNA were marginally increased in patients with pT3 or pT4 tumors compared to those with pT 2 or less. The levels of microbial 16S rDNA tended to increase in patients with higher pathologic tumor stage, Gleason score, and margin and lymph node involvements; but these differences did not reach significance.

**Conclusion:** The plasma 16S rDNA levels increased in patients with PCa who have biochemical recurrence and 16S rDNA levels were higher in patients with higher-grade PCa.

## Introduction

Prostate cancer (PCa) is one of the most common causes of morbidity and mortality in men. Several risk factors for PCa have been described, including environmental factors (e.g., infection and microbiome), host immunological parameters (e.g., anti-tumor immunity and inflammation), genetic effects, and behavioral factors (1–3).

Prostate cancer may develop in the setting of chronic inflammation, with pleomorphic inflammatory cell infiltrates and activation of toll-like receptors (TLRs) (4). Heightened inflammation has been observed in PCa tissues relative to benign prostate tissues (5), indicating the link between chronic inflammation and PCa biology. Chronic inflammation may be come from bacterial colonization or infection of the prostate gland or urethra, and through exposure to bacterial product translocation from the mucosa. However, in many patients, a source of chronic infection and inflammation is not apparent. Bacterial products are shown to mediate cancer development, and their inhibitors have been applied to treat certain cancers (e.g., colon cancer) (6). Nevertheless, the effects of the long-term, repeated low-dose exposure to bacterial products on PCa development or progression remain unknown.

Increasing evidence shows that the microbiome affects oncogenesis and tumor progression. For example, enrichment of *Porphyromonas gingivalis* is associated with the risk of pancreatic cancer in human; enrichment of *L. iners* is associated with the risk of cervical cancer in human (7, 8). Notably, distinct Bacteroides species in the gut is associated with the efficacy of CTLA-4 blockade used as cancer immunotherapy on melanoma patients as well as in mice (9). However, studies on microbiome and PCa in human are limited. Although *Escherichia coli* and *Enterococcus* species are known to promote prostatitis, there is no clear clue on the role of prostatitis in prostate cancer (10). In addition, the urethra runs through the center of the prostate, which is in front of the rectum; urinary microbiome may serve as a route of prostate exposure to bacteria or bacterial products contained in or passing through the urethra due to urinary anatomical proximity and play a role in prostate cancer prevalence (11).

The bacterial 16S ribosomal DNA (rDNA) assay can analyze 90% of bacterial strains, including gram-positive and gram-negative bacteria (12). In the current study, we have evaluated the magnitude of microbial translocation by total bacterial 16S rDNA in plasma using quantitative PCR (qPCR).

## Materials and Methods

### Study Subjects

All plasma samples were obtained from the Medical University of South Carolina (MUSC) Biorepository; which were originally obtained from preoperative PCa patients undergoing prostatectomy. All samples were de-identified; therefore, this study was not belonging to human subject study and consent was not required. Pathology and follow-up clinical data were obtained from existing medical records. At least two measurements of blood PSA level above 0.2 ng/mL after prostatectomy is defined as PCa relapse.

### Isolation of Microbial DNA From Plasma Samples

Microbial DNA was extracted from 400 μL of EDTA-anticoagulated plasma and the endotoxin-free water control using the QIAamp UCP Pathogen Mini Kit (Qiagen, Valencia, CA, USA) according to the manufacturer's instruction.

### Quantitative Polymerase Chain Reaction (PCR) for Measurement of Bacterial 16S rDNA

The method was described in our previous studies (12). Briefly, a 20 μL amplification reaction consisted of 10 μL of 2x Perfecta qPCR ToughMix (Quanta, Gaithersburg, MD), 0.3 μmol/L forward and reverse primers, 0.175 μmol/L probe (338 P: 5′-FAM-GCTGCCTCCCGTAGGAGT-BHQ1-3′), and 5 μL of the template plasma DNA. Degenerate forward (8 F: 5′-AGTTTGATCCTGGCTCAG-3′) and reverse (515 R: 5′-GWATTACCGCGGCKGCTG-3′) primers were used to amplify DNA templates encoding 16S rRNA. The DNA was amplified in duplicate, and mean values were calculated by subtracting bacterial 16S DNA values in the water control. A standard curve was created from serial dilutions of plasmid DNA containing known copy numbers of the template. The reaction conditions for amplification of DNA were 95°C for 5 min, followed by 40 cycles at 95°C for 15 s, and 60°C for 1 min.

### Plasma Levels of Testosterone and PSA

Plasma level of PSA was measured using high-sensitivity ELISA kits (R&D, Inc, Minneapolis, MN, USA) according to the manufacturer's instruction.

### Statistical Analysis

Statistical analysis was performed by GraphPad Prism 6.0 (GraphPad, San Diego, USA) using the Mann-Whitney's *U*-test (unpaired) and Spearman correlation test. *P* ≤ 0.05 were considered statistically significant.

## Results

### Plasma Levels of Microbial Translocation in Healthy Controls and PCa Patients

**Figure 1:**
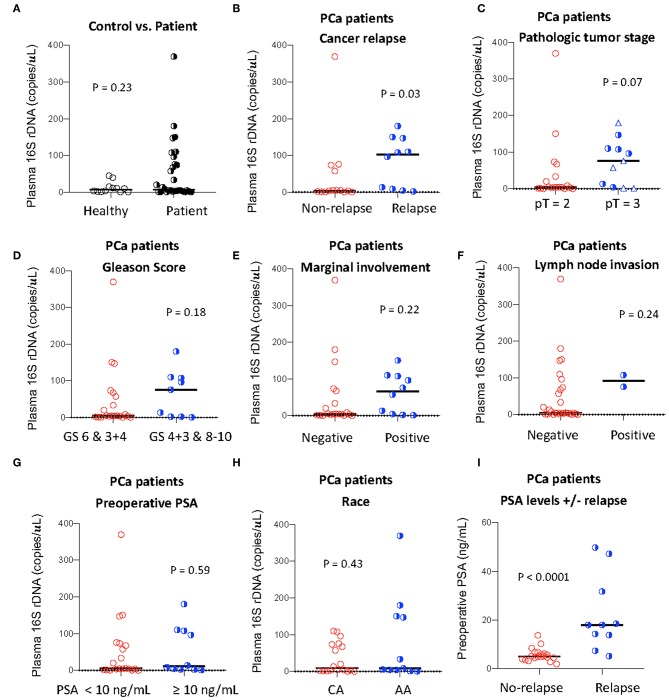
Plasma levels of total bacterial 16S rDNA and its association with PCa progression. Plasma level of total bacterial 16S rDNA was measured by qPCR in healthy controls and PCa patients **(A)**, in patients with or without relapse **(B)**, with pT2 or pT3 (among pT = 3, circles: pT = 3A; triangles: pT = 3B, **C**), with high or low Gleason scores (GS, **D**), with or without marginal involvement **(E)**, with or without lymph node invasion **(F)**, with high or low preoperative PSA levels **(G)**, and with Caucasian (CA) or African American (AA) **(H)**. Preoperative PSA levels in patients with or without relapse **(I)**. Mann-Whitney U (unpaired).

Clinical characteristics of patients and controls are shown in Table 1. The plasma levels of total bacterial 16S rDNA were similar in healthy control men and PCa patients (*P* = 0.23, Figure 1A).

**Table 1:** Clinical characteristics of PCa patients.

	**Non-relapse (*n* = 17)**	**Relapse (*n* = 10)**	***P*-value**
Age 61	(54.5–64)	64 (60.5–69)	0.17
Race AA/CA (%AA)	35.3	40	>0.99
**Pathologic tumor stage**
pT2	1/17 (5.9%)	0/10 (0%)	>0.99
pT3	3/17 (17.6%)	7/10 (70%)	0.01
**Tumor pathology**
≤ Gleason score 3+4	15/17 (88.2%)	2/10 (20%)	0.0007
≥Gleason score 4+3	2/17 (11.8%)	8/10 (80%)	0.0007
Margin+	3/17 (17.6%)	7/10 (70%)	0.01
Lymph node invasion	1/17 (5.9%)	1/10 (10%)	>0.99

### Association of Plasma Microbial Translocation and PCa Relapse After Prostatectomy

To investigate the association of the magnitude of microbial translocation and PCa progression, we first have stratified patients to cancer relapse after 2 years of prostatectomy. Among 27 PCa patients, 10 PCa patients had relapsed within 2 years or less after prostatectomy. Five of these patients relapsed within 100 days after prostatectomy. Patients with relapse had increased plasma 16S rDNA levels compared to patients without relapse; the median and interquartile of plasma 16S rDNA levels (copies/μL) were 4.38 (1.35–62.5) and 102.1 (7.50–157.8) in patients without relapse or with relapse, respectively (*P* = 0.03, Figure 1B). Furthermore, patients with relapse within 100 days after prostatectomy tended to have increased plasma microbial translocation, compared to patients with relapse more than 100 days after prostatectomy, but the difference did not achieve significance (*P* = 0.22).

### Plasma Microbial Translocation and High-Risk Pathologic Features

We next evaluated the associations between plasma levels of 16S rDNA and PCa progression and risk markers for recurrence. Plasma 16S rDNA levels were marginally increased in patients with pathologic tumor stage of pT3 compared to pT2 (*P* = 0.07, Figure 1C). Furthermore, plasma 16S rDNA levels tended to increase in patients with margin involvement, lymph node invasion, and Gleason score of 4+3 and 8–10, compared to their controls, but these differences did not achieve significance (Figures 1D–F). There was no difference in levels of plasma microbial translocation in patients with preoperative PSA levels lower than 10 ng/mL compared to those with PSA equal or more than 10 ng/mL, and in patients of African and American or Caucasian race (Figures 1G,H). Consistent with previous studies (13), a higher preoperative PSA value was significantly associated with patients with relapse after prostatectomy (*p* = 0.005, Figure 1I).

## Discussion

Long-term repeated bacterial stimulation may affect the expression and activities of TLR4, the receptor for bacterial lipopolysaccharide (LPS). Furthermore, increased TLR4 expression and responsiveness has been shown in PCa cells compared to non-cancer prostate cells (4). Thus, bacteria or bacterial products may contribute to PCa oncogenesis and pathogenesis via the TLR4 or other TLR cell signaling pathway. Indeed, reduced PC3 cell migration and invasion have been observed after TLR4 knockout (14). In addition, treatment of a TLR4 ligand (Peroxiredoxin-1) mediates PCa cell growth in a murine cancer model (15). Moreover, microbial mediated cytokines IL-6, IL-8 and IL-10 have been shown to promote PCa development and disease progression (16).

Chronic bacterial colonization in the prostate may result from the retrograde translocation of bacteria from the urethra (11). Other possibilities of bacteria or bacterial product translocation are mucosal sites at the rectum, oral cavity, lung, and vagina or systemic dissemination of infection from distant foci (17, 18). These studies and results from our study suggest that systemic bacterial translocation may contribute to PCa relapse within 2 years. A proximate mechanism could involve activation of the innate immune system or other inflammatory processes.

Cancer-related immunity plays a role in PCa development and progression. T regulatory cells, tumor-associated macrophages, and myeloid-derived suppressor cells mediate local immune inhibitory environment (2, 19); which may decrease anti-tumor immunity, or promote cancer cell transformation (4, 20). Thus, immune inhibitory cytokines and chemokines and decreased anti-tumor immunities in the local microenvironment may promote PCa cell survival, activation, proliferation, invasion, and metastasis (20). Further studies need to investigate the role of bacterial translocation in altering systemic inflammation, anti-tumor immunities, and PCa biology.

The main limitation of our study is the small size, which prevents us to draw any further conclusion. In addition, potential confounders were not controlled in the current study. In summary, we found that plasma 16S rDNA levels increased in patients with PCa who have biochemical recurrence and 16S rDNA levels were higher in patients with higher-grade PCa.

## Significance

Our study indicates that preoperative level of systemic microbial translocation may play a role in prostate cancer (PCa) outcomes, and treatment targeting mucosal barrier may prevent PCa relapse after prostatectomy.

## Data Availability Statement

The datasets generated for this study are available on request to the corresponding author.

## Author Contributions

TO and ZZ performed experiments and wrote the first version of the manuscript. BZ, DT, ML, and WJ designed the study, analyzed the data, and revised the manuscript.

### Conflict of Interest

The authors declare that the research was conducted in the absence of any commercial or financial relationships that could be construed as a potential conflict of interest.
